# Altered B cell phenotype and CD27+ memory B cells are associated with clinical features and environmental exposure in Colombian systemic lupus erythematosus patients

**DOI:** 10.3389/fmed.2022.950452

**Published:** 2022-09-06

**Authors:** Carolina Hurtado, Diego Fernando Rojas-Gualdrón, Rodrigo Urrego, Kevin Cashman, Elsa María Vásquez-Trespalacios, Juan Camilo Díaz-Coronado, Mauricio Rojas, Scott Jenks, Gloria Vásquez, Ignacio Sanz

**Affiliations:** ^1^School of Medicine, Universidad CES, Medellín, Colombia; ^2^School of Graduate Studies, Universidad CES, Medellín, Colombia; ^3^Group INCA-CES, School of Veterinary Medicine and Zootechnic, Universidad CES, Medellín, Colombia; ^4^Lowance Center for Human Immunology, Department of Medicine, Emory University, Atlanta, GA, United States; ^5^Group of Clinical Information, Artmedica IPS, Medellín, Colombia; ^6^Grupo de Inmunología Celular e Inmunogenética, Universidad de Antioquia, Medellín, Colombia; ^7^Unidad de Citometría de Flujo, Universidad de Antioquia, Medellín, Colombia

**Keywords:** autoimmunity, B lymphocytes, systemic lupus erythematosus, lupus nephritis, IFI44L, solvents, occupational exposure

## Abstract

**Background:**

B lymphocytes are dysregulated in Systemic Lupus Erythematosus (SLE) including the expansion of extrafollicular B cells in patients with SLE of African American ancestry, which is associated with disease activity and nephritis. The population of Colombia has a mixture of European, Native American, and African ancestry. It is not known if Colombian patients have the same B cell distributions described previously and if they are associated with disease activity, clinical manifestations, and environmental exposures.

**Objective:**

To characterize B cell phenotype in a group of Colombian Systemic Lupus Erythematosus patients with mixed ancestry and determine possible associations with disease activity, clinical manifestations, the DNA methylation status of the IFI44L gene and environmental exposures.

**Materials and methods:**

Forty SLE patients and 17 healthy controls were recruited. Cryopreserved peripheral B lymphocytes were analyzed by multiparameter flow cytometry, and the DNA methylation status of the gene IFI44L was evaluated in resting Naive B cells (rNAV).

**Results:**

Extrafollicular active Naive (aNAV) and Double Negative type 2, DN2 (CD27− IgD− CD21− CD11c+) B cells were expanded in severe active patients and were associated with nephritis. Patients had hypomethylation of the IFI44L gene in rNAV cells. Regarding environmental exposure, patients occupationally exposed to organic solvents had increased memory CD27+ cells (SWM).

**Conclusion:**

aNAV and DN2 extrafollicular cells showed significant clinical associations in Colombian SLE patients, suggesting a relevant role in the disease’s pathophysiology. Hypomethylation of the IFI44L gene in resting Naive B cells suggests that epigenetic changes are established at exceedingly early stages of B cell ontogeny. Also, an alteration in SWM memory cells was observed for the first time in patients exposed to organic solvents. This opens different clinical and basic research possibilities to corroborate these findings and deepen the knowledge of the relationship between environmental exposure and SLE.

## Introduction

Systemic lupus erythematosus (SLE) is a highly heterogenic autoimmune disease (1). Symptoms and organ involvements vary from patient to patient, and they can virtually affect any system in the body (2). Patients also have different grades of disease activity, characterized by periods of relapses and flares that repeat over time (3). This clinical variability leads to different treatment regimens according to specific clinical characteristics like the presence of renal involvement (4). Ethnicity also influences SLE. Latin-American patients have the second-highest frequency of SLE, after African Americans (5). Data from the cohort GLADEL, which enrolled patients from 9 Latin American countries, have also described specific clinical characteristics associated with this population (6). They found that African Latin American and Mestizo (mixed European and Amerindian ancestry) patients had more severe disease and a higher frequency of renal disease when compared to Caucasian patients (7). Also, Mestizo patients have a worse prognosis (8) and present renal activity earlier in the condition (9).

Among this heterogeneity, a common factor in all SLE patients is the dysregulation of B cells, making them a vital treatment target (10). These B cells abnormalities account for frequency changes on subsets and functional alterations like autoreactivity. While the decrease in the frequency of CD27^+^ IgD^+^ Unswitched B cells (USM) seems to be a widespread change in most SLE patients (11), other changes, such as an expansion of recently described extrafollicular effector double negative type 2 (DN2) and activated naive (aNAV) B cells, are specific to specific patient subgroups such as patients of African American ancestry with active disease status and renal involvement (12). The population of Colombia is highly diverse and it has a mixture of European, Native American, and African ancestries (13). It is unknown if Colombian patients have the same B cell distributions previously described and if they are associated with clinical variables; therefore, we aimed to characterize it in the present study.

Another factor playing an essential role in SLE is epigenetics, which significantly changes DNA methylation, as these changes respond to some environmental exposures (14). Interestingly, ethnicity can also influence DNA methylation (15). Differences have also been described in the methylation status of certain genes, such as hypomethylation of specific genes such as PLSCR1, IFIT1, and IFI44L, significant for patients of African American origin, not for European American patients (16). The methylation status of the promoter of the IFI44L gene was also proposed as a diagnostic marker. This was validated in 2 cohorts of patients of Chinese origin. However, when validated in a cohort of European patients, sensitivity and specificity dropped (17). This could indicate that some of these new biomarkers could work better according to ethnicity. As the methylation status of IFI44L could also variate by race, finally, we also examined it in a cohort of Colombian patients of mixed ethnicity.

On the other hand, B cell dysregulation could also be caused by environmental factors like the Epstein Bar virus (18). In addition, murine models exposed to trichloroethylene (TCE), an organic solvent used in degreasers, showed an acceleration of the autoimmune response, deposition of immune complexes, increased antibody titers, and manifestations of activity (19). Moreover, while alterations in T cells have been reported on occupational organic solvent exposure (20), neither animal models nor exposed human studies’ have explored possible effects on B cell subpopulations. Indeed, very little is known regarding the association between environmental and occupational factors, B cell subpopulations’ frequency, and its relation to SLE.

Therefore, we characterized the B cell phenotype in a group of Colombian SLE patients with mixed ancestry and explored associations with disease activity, clinical manifestations, the DNA methylation status of the IFI44L gene, and environmental exposures.

## Patients and methods

### Patients and healthy controls

Systemic lupus erythematosus patients were recruited in an outpatient care center, Artmedica, a reference center for autoimmune disease treatment in Medellín, Colombia, from April to July 2018. Inclusion criteria were patients over 18 years old that fulfilled > 4 criteria of the modified ACR criteria 1982 at the diagnosis by the rheumatology service. Exclusion criteria included drug-induced SLE, polyautoimmunity, recent treatment with rituximab, infection, and cancer history. Healthy Controls (HC) were selected by gender and age, to achieve a composition similar to that of the patients and the same exclusion criteria were applied. This study received approval from the Ethics Committee on Research on Human Beings of CES University with the record number 118, and all participants signed informed consent. Also, a cohort of European American patients was included as a control group for some analysis, recruited through the Lowance Center for Human Immunology, Emory University, United States. The Emory Institutional Review Board (58515 and 58507) approved the study and all participants signed informed consent.

### Disease activity classification and clinical data

The Mexican version of SLEDAI, Mex-SLEDAI score (21), previously validated (22), was used to classify activity. To classify patients on moderate and severe activity (23), score cut-off values were: inactive Mex-SLEDAI < 2, moderate Mex-SLEDAI 3–7, and severe disease activity Mex-SLEDAI ≥ 8. Clinical records were reviewed to obtain information on ACR criteria at the diagnosis, history of organ damage, current treatment, autoantibodies titers, and exclusion criteria.

### Cell isolation, freezing, and cell thawing

Blood samples were drawn by cubital fossa venipuncture, and EDTA tubes were used for blood sample collection. Peripheral blood mononuclear cells (PBMC) were isolated by density gradient using Histopaque^®^ (Ref 10771 Sigma-Aldrich Co. LLC, Darmstadt, Germany). Freezing media was prepared with Fetal Bovine Serum (FBS ThermoFisher Scientific, Waltham, MA, United States), and 10% of Dimethylsulfoxide (DMSO) and cells were stored in liquid nitrogen at −196°C until thawing.

For cell thawing, cells were warmed at 37°C in the bath, then washed with 37°C RPMI (Cat Number MT-10043CV, ThermoFisher Scientific, Waltham, MA, United States) supplemented with 20% FBS. After two washes with phosphate buffered saline (PBS, ThermoFisher Scientific, Waltham, MA, United States), trypan blue in a concentration of 1:10 was added to the cells. Finally, a counting and viability test with trypan blue was performed with an automated cell counter (Bio-Rad Hercules, CA, United States).

### Flow cytometry and cell sorting

As Kaminski et al. (24) described, multiparametric Flow Cytometry was used. Cells were characterized using CD3 (Clone SP34-2, BD Biosciences NJ, United States), CD19 (Clone SJ25C1 BD Biosciences), CD38 (Clone HIT2, Ebioscience, San Diego, CA, United States), CD27 (Clone L128, BD Biosciences), IgD (clone IA6-2, BD Biosciences), CD11c (Clone B-ly6, BD Bioscience), CD21 (Clone B-ly4, BD Bioscience), and CXCR5 (Clone 51505, R&D) markers. Also, we excluded CD38^++^ CD27^++^ Plasma Cells and dead cells with a Fixable Viability Dye eFlour 506 (Thermo Fisher, Scientific, Waltham, MA, United States). Anti-VH4.34 antibody (clone 9G4) was also used. The markers IgD+ CD27− CXCR5+ CD11c− were used for sorting resting Naive B cells. For compensation, we used beads stained with each fluorochrome. A BD LSR II cytometer was used for reading and BD FACS Aria II SORP Cell Sorter. The B cell binding index (BCB) was calculated as described in Jenks et al. (25), using the following formula [Resting naive 9G4 + median fluorescence intensity]/[SWM 9G4 + median fluorescence intensity]. FlowJo™ v9/10 Software (BD Life Sciences) was used for cytometric analysis.

### Dimensionality reduction analysis

All dimensionality reduction analyses were performed using FlowJo™ v9/10 Software (BD Life Sciences). We first concatenated all files in one and then ran the UMAP analysis plugin. Next, to identify the ideal number of clusters, the X-Shift plugin was used, and, finally, to visualize the marker’s expression levels on each cluster, we ran Cluster Explorer Plugin.

### DNA extraction and methylation analysis IFI44L

Genomic DNA from patients was subjected to bisulfite conversion to study the methylation status of a region from gen IFI44L. DNA was extracted with AllPrep DNA/RNA Micro Kit (QIAGEN) according to manufacturer’s instructions. Briefly, cells were disrupted with Buffer RLT Plus, homogenized by vortexing, and the lysate was transferred to the DNA spin column to centrifuge. After washing with buffers AW1 and 2, DNA was eluted with Buffer EB, and quality was evaluated with a NanoDrop Spectrophotometer. DNA was stored at −80°C until its use. The methylation status of two CpG sites within the IFI44L promoter, Site1 (Chr1: 79 085 222) and Site2 (Chr1: 79 085 250; cg06872964), were then analyzed by pyrosequencing, and specific primers were used to amplify the target promoter fragment: forward 5′- GTAGTTTTATTTAGTTTTGGGGTATTTG-3′; reverse 5′- CCCCACCCCTTATAAATCCAATACTATCAC-3′ tagged with biotin at 5′ end. The PCR product was sequenced by pyrosequencing with the specific probe: 5′- AGTAAGGAAGTTAGGAGAATA-3′. All samples were sequenced using PyroMark Q48 Autoprep (QIAGEN) in Macrogen CO. (Seoul, South Korea).

### Organic solvent exposure survey

To determine environmental exposure, we used a questionnaire-based instrument constructed to characterize relevant environmental exposures, designed explicitly for SLE patients, in a previous study (26). This instrument allowed us to define which patients and HC were occupationally exposed to organic solvents.

### Statistical analysis

Descriptive analysis was carried out using frequencies and means and standard deviation or median with 95% CI. Mann–Whitney test or Welch test was used for comparison between two independent samples. Multivariate Analysis of Variance (MANOVA) was used to analyze differences for three or more groups, as suggested by Genser et al. for statistical analysis of immunological data like cell subsets (27). Associations between frequencies of B cell subpopulations, disease activity, clinical characteristics, and ethnicity were analyzed using multivariate linear regression, taking the logarithms of the subpopulation frequencies as dependent variables and adjusting for clinical characteristics. In addition, a hierarchical cluster analysis was performed and presented as a heatmap.

The level of significance was defined at 0.05. Statistical analysis was executed on Stata 15.1 (College Station, TX, United States) and the figures and some analysis on GraphPrism 8.1.2.

## Results

### Clinical and demographic characteristics

Forty SLE patients and 17 HC were evaluated; 90% were female, with a mean of 39 years of age (SD 15). The distribution of SLE patients by activity comprised 10 active and 30 inactive patients classified according to MEX-SLEDAI; 80% of them had joint and renal involvement and were treated with different immunosuppressant regimes. Demographic characteristics are depicted in Supplementary Table 1.

### Severe active systemic lupus erythematosus patients have even higher aNAV and DN2 cell frequencies than moderate or inactive systemic lupus erythematosus patients

**FIGURE 1:**
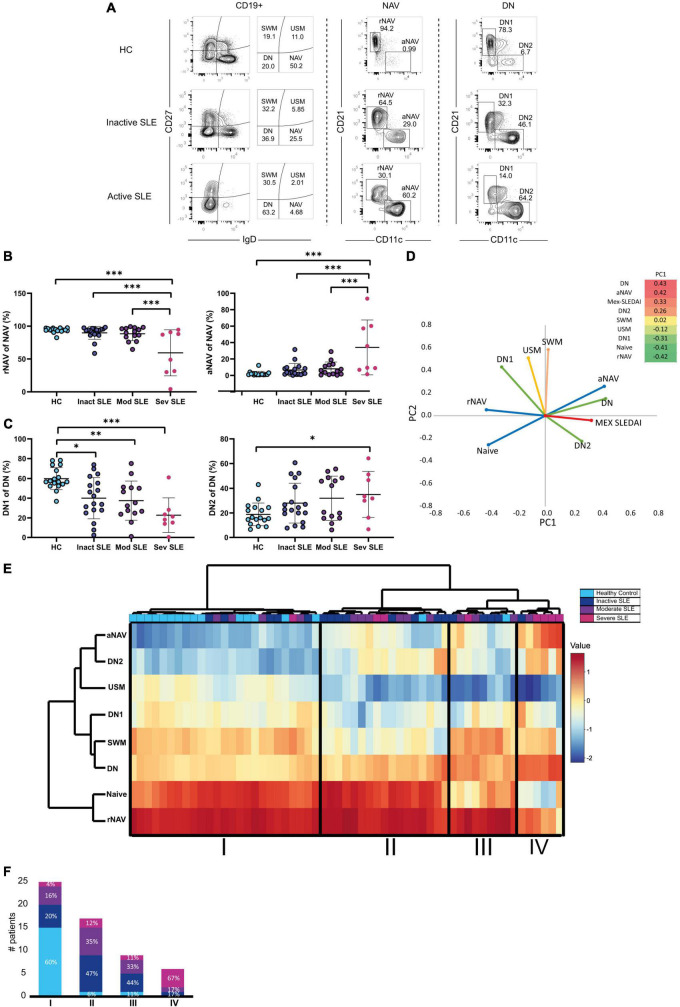
B cell subsets and disease activity. **(A)** Gating strategy for B cell subsets analysis. Mex-SLEDAI scores for an inactive patient were 3, and 9 for an active patient. **(B)** Active (aNAV) and resting (rNAV) B cells in Healthy Controls (clear blue), Inactive (dark blue), Moderate (purple), and Severe (dark pink) active SLE patients. Multivariate Analysis of Variance (MANOVA) was used for analysis and means, and SD are depicted in the figures. **(C)** DN1 and DN2 cells in Healthy Controls (clear blue), Inactive (dark blue), Moderate (purple), and Severe (dark pink) active SLE patients. Multivariate Analysis of Variance (MANOVA) were used for analysis and means, and SD are depicted in the figures. **(D)** Principal Component Analysis of B cell subsets. **(E)** Hierarchical cluster analysis of B cell subpopulations. **(F)** Percentage of patients with severe activity, moderate activity, inactive and healthy controls in each detected cluster. *P ≤ 0.05; **P ≤ 0.01; ***P ≤ 0.001.

B cell frequency was determined according to three groups of disease activity: inactive, moderate activity, and severe activity groups (described in Section “Patients and methods”). To characterize B cell subsets, CD19^+^ B cells were classified on IgD and CD27 markers into Switched Memory CD27^+^IgD^–^ (SWM); Unswitched Memory CD27^+^IgD^+^ (USM); Naive CD27^–^IgD^+^ (NAV); and Double Negative CD27^–^IgD^–^ (DN) B cells. NAV cells were further classified into resting Naive CD21^+^ CD11c^–^ (rNAV) and active Naive CD21^–^ CD11c^+^ (aNAV). DN was also divided into DN1: CD21^+^ CD11c^–^ and DN2: CD21^–^ CD11c^+^. The strategy of analysis and illustrative expansion of aNAV and DN2 cells on active patients is shown in Figure 1A.

Severe active patients had the highest frequency of aNAV cells, compared to moderate and inactive patients and HC (34.1 vs. 8.07 *p* = 0.0001) (Figure 1B). DN2 cells were also increased on severe patients compared with HC (15.6 vs. 2.9 *p* = 0.002), but the difference was not significant with moderate or inactive SLE patients (35.05 vs. 31.9, respectively *p* = 0.998) (Figure 1C).

Regarding memory B cells, USM cells decreased in severe active patients compared to HC (6.9 vs. 1.7 *p* = 0.0001) but did not differ between moderate or inactive patients (1.77 vs. 2.93 *p* = 0.915), and this contrasts with SWM cells that did not vary by activity (Supplementary Figure 1B).

To explore further this association of extrafollicular B cells and disease activity, we performed Principal Component Analysis (PCA), and it explained 71% of the variance and Principal Component 1 (PC1) clustered together aNAV, DN2, and DN subsets to Mex SLEDAI score (Figure 1D). In addition, a heat map was made to picture the weight of each variable in PC1. This is in line with the association of aNAV and DN2 cells to the active status of the disease described in Figures 1B,C.

Hierarchical cluster analysis allowed group patients according to their activity status in Clusters II, III, and IV and to discriminate them from HC in an independent Cluster in Cluster I (Figure 1E). Indeed, most HC was grouped in Cluster I, unlike patients with severe activity mainly grouped in Cluster IV. Clusters II and III consisted mainly of inactive or moderately active patients (Figure 1F).

Changes in cell frequencies become more relevant in the context of functionality. For this reason, we determined the frequency of autoreactivity in B cell subsets by calculating the frequency of 9G4 positive VH4.34 antibodies in the subsets (28–30).

Patients tended to have more autoreactive cells in each of the B cell subpopulations, and this difference was significant for DN2 9G4+ cells compared to HC (0.7–1.4 *p* = 0.0303) (Supplementary Figure 2A). Patients, but not HCD, also had B cell binding 9G4+ antibodies (Supplementary Figure 2B), which suggests a break in tolerance and the production of autoreactive antibody secreting cells (31).

### Dimensionality reduction analysis confirmed clusters of B cells with extrafollicular markers overexpressed in severe active patients and identified possible new related B cell subsets

**FIGURE 2:**
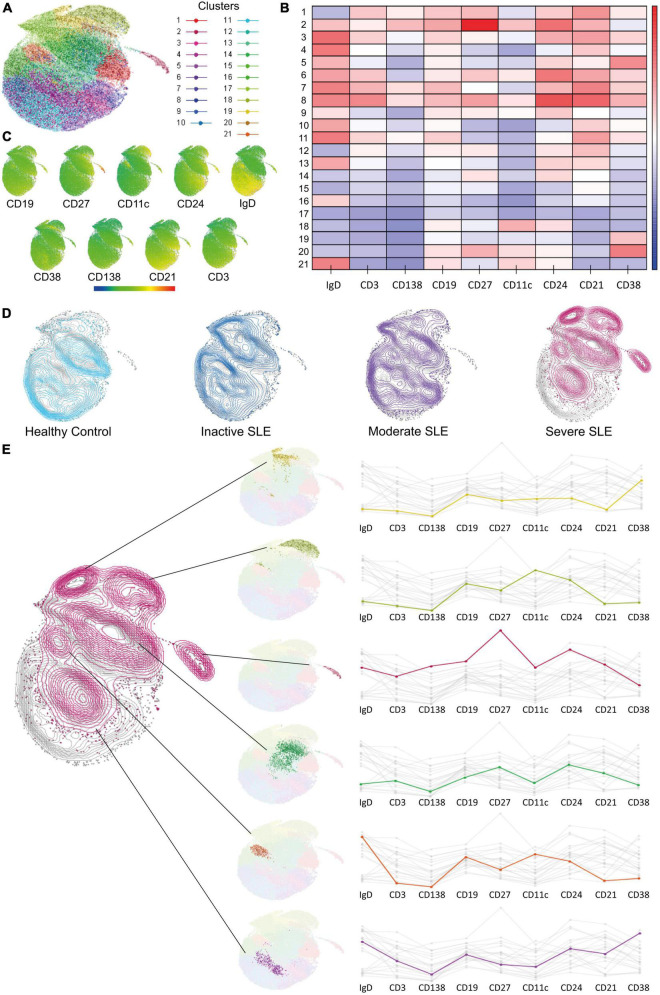
Dimensionality reduction analysis to identify new B cell subsets. **(A)** UMAP analysis for dimensionality reduction and identified clusters. **(B,C)** Heat map showing marker’s expression levels for each cluster. **(D)** Comparison of cluster’s expression in Healthy Controls (clear blue), Inactive (dark blue), Moderate (purple), and Severe (dark pink) active SLE patients. **(E)** Clusters with a differential expression when comparing between HC and patients with severe active SLE patients and its markers.

Traditional gating strategies to analyze flow cytometry data could have a weakness in detecting subsets with exceptionally low frequencies; also, operator bias should be considered. Dimensionality reduction analysis can overcome both (32). Therefore, we performed a UMAP analysis and used X-Shift and Cluster explorer plugins of Flowjo to further exploration. Twenty-one clusters were detected (Figure 2A), each with a differential expression of markers (Figures 2B,C). Then we compared the expression of each cluster according to disease activity and found a gradual and progressive change in the expression of each cluster as the level of activity increased (Figure 2D). Two of the overexpressed clusters in severe active patients had markers compatible with aNAV and DN2, which is in line with Figure 1. One cluster has memory compatible markers, and the other two have Naive compatible markers, and both could represent new B cell subsets associated with severe activity (Figure 2E). However, this should be confirmed in further research.

### Extrafollicular B cell subsets are associated with clinical features like nephritis

**FIGURE 3:**
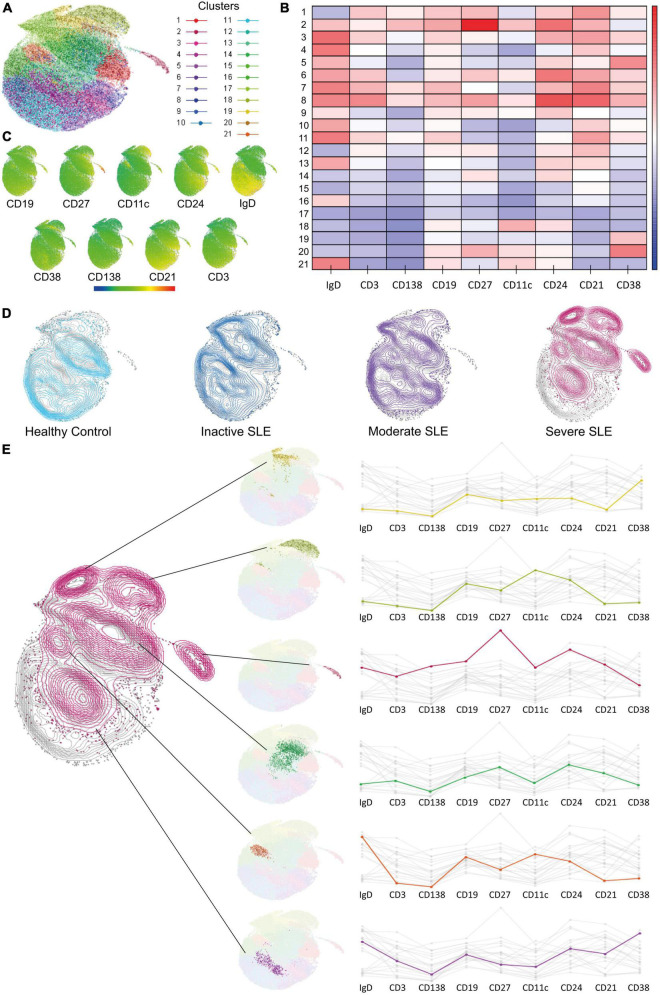
B cell subsets distribution on patients and clinical associations. **(A)** B cell subpopulations associated with nephritis. Mann–Whitney *T*-test was used for analysis, and median and 95% CI are depicted in the figures. **(B)** B cells are associated with a history of hematologic involvement. Mann–Whitney *T*-test was used for analysis, and median and 95% CI are depicted in the figures. **(C)** Association of USM cells in patients with a history of thrombosis Mann–Whitney *T*-test was used for analysis, and median and 95% CI are depicted in the figures. **(D)** B cells subsets associated to low C3 levels. Mann–Whitney *T*-test was used for analysis, and median and 95% CI are depicted in the figures. **(E)** Clinical characteristics associated with clusters identified in Figure 1E: renal activity and high DN2 values; the Chi-square test were used for analysis. **(F)** Ethnicity and DN1 and DN2 Cells. AC, African Colombian; MC, Mestizo Colombian. Mann–Whitney *T*-test was used for analysis, and median and 95% CI are depicted in the figures. **(G)** Ethnicity and rNAV and aNAV Cells. Mann–Whitney *T*-test was used for analysis, and median and 95% CI are depicted in the figures. **(H)** Ethnicity and DN1 and DN2 Cells in Colombian and European American SLE patients. Mann–Whitney *T*-test and Kruskal Wallis was used for analysis, and median and 95% CI are depicted in the figures. *P ≤ 0.05; **P ≤ 0.01; ***P ≤ 0.001.

We next explored B cell subsets distribution in patients with nephritis. Patients with history of nephritis also had an increase in aNAV (17.8 vs. 6.2; *p* = 0.0410), DN (26.6 vs. 19.4; *p* = 0.0359) and DN2 (9.3 vs. 4.9; *p* = 0.0295) cells compared to patients without nephritis (Figure 3A).

Another widespread manifestation of SLE is hematological involvement, including leukopenia, lymphopenia, hemolytic anemia, and thrombosis events. Because only leukopenia, anemia, and thrombocytopenia score on Mex-SLEDAI we grouped all history of hematological involvement to examine the association with B cell subsets. We found an association of increased aNAV (9.3 vs. 2.4; *p* = 0.0068), decreased rNAV (94.5 vs. 86.3; *p* = 0.0139) and increased DN cells (27.20 vs. 19; *p* = 0.0247) (Figure 3B) with hematological involvement.

### Lower USM cells are associated with a previous thrombosis event

After analyzing all events together, each type of hematological compromise was also analyzed, and, interestingly, patients with a previous thrombosis event had a reduction in USM cells (2.9 vs. 1.2; *p* = 0.0256) (Figure 3C). This is an exploratory observation, given the limitation in the number of patients with thrombosis (*n* = 5). However, since it has previously been thought that the loss of USW is a general feature of autoimmunity (33), this is a novel finding that future research should explore further.

### Anti Sm, anti RNP, and low C3 were associated with increased aNAV and DN2 cells

The presence of autoantibodies is an important clinical feature for diagnosing SLE; therefore, we determined possible correlations of autoantibodies to B cell subsets distributions. Anti Ro, Anti La, Anti Sm, and Anti RNP were obtained from clinical registers; of the 30 patients with a complete autoantibodies profile, 50% had positive Anti Ro and anti-RNP 36.6% were positive for Anti Sm, and 20% had Anti-La. Anti Sm was associated with increased frequencies of aNAV (10.9 vs. 2.4 *p* = 0.0172), DN (36.3 vs. 19 *p* = 0.0213), and DN2 (14 vs. 2.9 *p* = 0.0093) (Supplementary Figure 3A). Furthermore, Anti RNP was also associated to increased aNAV (8.4 vs. 2.02 *p* = 0.0190), DN (23.9 vs. 16.9 *p* = 0.0454), and DN2 (11.6 vs. 2.4 *p* = 0.0037) (Supplementary Figure 3B).

C3 and C4 consumption are useful in disease activity measurement and are part of the SLEDAI. Although the number of patients recorded was limited, it was observed that patients with low C3 levels had higher aNAV cell frequencies (19.9 vs. 5.6, *p* = 0.0213), DN (47.5 vs. 21.9, *p* = 0.0049) and DN2 (15.3 vs. 7.5, *p* = 0.0139) (Figure 3D). A low level of C3 values was < 80 mg/dL.

Also, more than 80% of the patients in cluster IV (Figure 1E) had renal activity and high DN2 values, and the percentages progressively increased from cluster I to IV (Figure 3E). The same phenomenon was observed with positive Sm and RNP antibodies (Supplementary Figure 3C).

### African Colombian patients had the highest frequencies of DN2 cells compared to Mestizo-Colombian and European American systemic lupus erythematosus patients

It has been reported that DN2 frequency is higher in patients of African American ancestry, and it is associated with disease activity and lupus nephritis in these patients (12). To characterize the association of ethnicity to DN2 cells, self-reported ethnicity by the patients was classified into African-Colombian and Mestizo—Colombian patients (Supplementary Table 1). Although it is a limited number of patients, African Colombian Patients had the highest frequencies of DN2 cells (52.3 vs. 22.8; *p* = 0.0091) (Figure 3F). They also had an increased frequency of aNAV cells (13.6 vs. 3.7; *p* = 0.0182) (Figure 3G).

Furthermore, Colombian SLE patients had higher DN2 cells than European American patients (9.45 vs. 4.84 *p* = 0.0479) (Figure 3H). When we compared among ethnicities, African-Colombian patients still had significantly highest DN2 cells than European American patients (*p* = 0.0232) (Figure 3H).

This led us to ask, of all these associations that we observed with clinical manifestations, which one will have more weight or greater strength of association in each subpopulation of B cells? A multivariate linear regression analysis was performed, which considers the interdependence of the subpopulations to establish if some clinical manifestations have more weight or greater strength of association in each subpopulation of B cells.

After adjusting, the factor with the greatest association strength for aNAV cells was severe activity (Log Coefficient 1.151 *p* = 0.037); for DN2, it was ethnicity (Log Coefficient 0.294 *p* = 0.047), and for USM, it was a history of thrombosis (Log Coefficient −0.593 *p* = 0.013) (Supplementary Table 2).

### Hypomethylation of promoter site 1 and 2 of IFI44L gene on rNAV B cells of systemic lupus erythematosus patients

**FIGURE 4:**
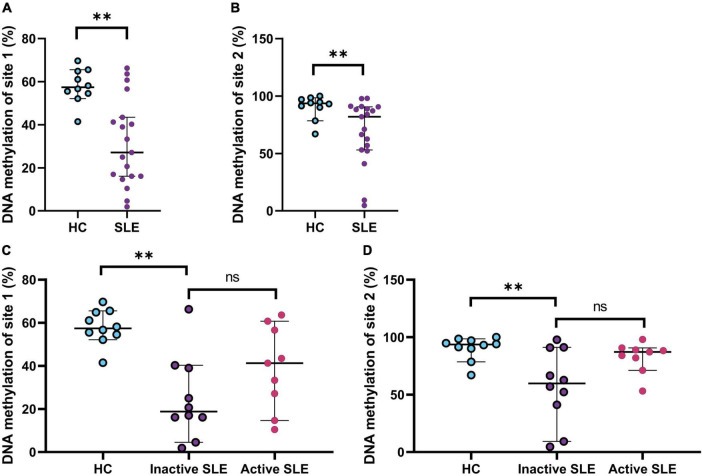
DNA methylation status of IFI44L gene on rNAV B cells. **(A)** Percentage of DNA methylation of site 1 on the promoter of the gene IFI44L in HC and SLE patients. Mann–Whitney *T*-test was used to analyze and median and 95% CI are depicted in the figures. **(B)** Percentage of DNA methylation of site 2 on the promoter of the gene IFI44L in HC and SLE patients. Mann–Whitney *T*-test was used to analyze and median and 95% CI are depicted in the figures. **(C)** Percentage of DNA methylation of site 1 on the promoter of the gene IFI44L on HC, inactive and active SLE patients. Kruskal Wallis with multiple comparisons used to analyze and median and 95% CI are depicted in the figures. **(D)** Percentage of DNA methylation of site 2 on the promoter of the gene IFI44L on HC, inactive and active SLE patients. Kruskal Wallis with multiple comparisons used to analyze and median and 95% CI are depicted in the figures. *P ≤ 0.05; **P ≤ 0.01; ***P ≤ 0.001.

DNA methylation state has long been recognized to play an important role in SLE physiopathology, and it occurs differentially on both T and B cell subsets (34). The hypomethylation of the IFI44L promoter has been proposed as a diagnostic biomarker in SLE (17). Therefore, to evaluate the DNA methylation status of IFI44L in B cells, we selected rNAV cells as this is the earliest mature B cell subpopulation that hallmark SLE epigenetic changes, shared with subsequently differentiated B cells subsets (35). SLE patients had hypomethylation of the site 1 (57.4 vs. 27.1; *p* = 0.0011) and site 2 (93.6 vs. 82.02; *p* = 0.0057) of the promoter of IFI44L gene (Figures 4A,B) (SLE *n* = 19; HC *n* = 10). Also, Inactive patients had the lowest DNA methylation percentage, especially on-site 1, compared to active patients; however, this was not significantly different (18.8 vs. 41.2; *p* = 0.1564) (Figures 4C,D). We also analyzed for different methylation percentages in patients with DN2 cells with high or low frequencies, but there were no differences (Supplementary Figure 4).

### Systemic lupus erythematosus patients exposed to organic solvents had higher frequencies of SWM cells

Given the relevance of environmental exposure in lupus, we wanted to know if environmental events were associated with B cells’ distributions. To answer this question, we constructed an instrument to characterize exposure to relevant environmental factors for SLE patients (26); this allowed us to classify patients based on exposure to organic solvents. The most used solvent was degreasers for cleaning in patients and HC, followed by ketones such as nail polish remover. Finally, 46% of the patients used protective equipment and none of the HC (Supplementary Table 3).

It has been reported that exposure to solvents can cause lymphopenia (36), but patients exposed to Organic Solvents (OS) did not have fewer total lymphocytes or CD19+ B cells than non-exposed individuals in this study (Supplementary Figure 5).

**FIGURE 5:**
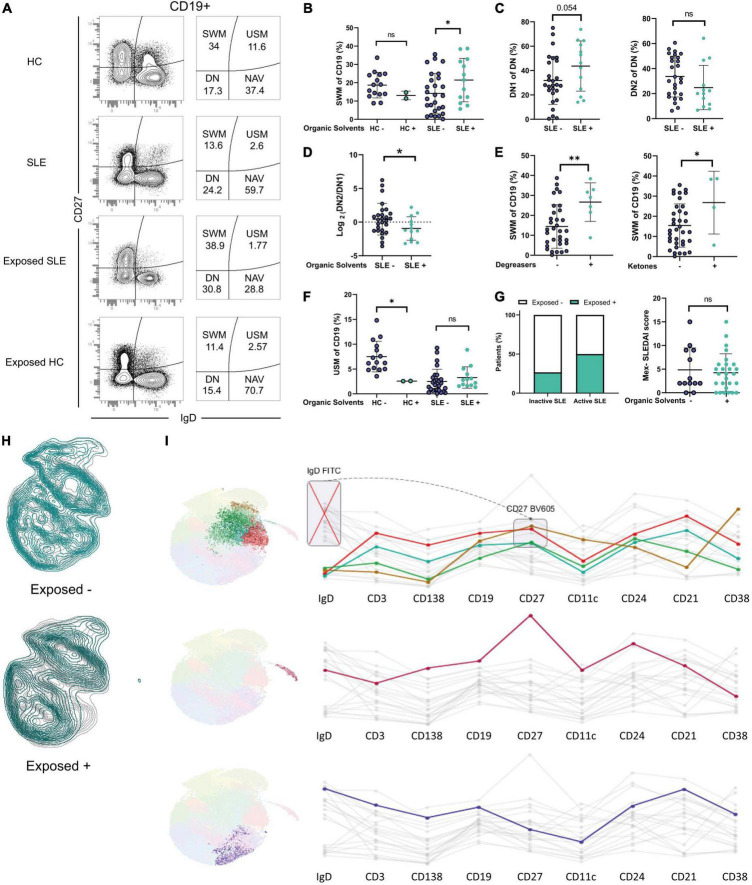
Distribution of B cells in patients and healthy occupationally exposed to Organic Solvents. **(A)** Representative dot plots showing increased SWM cells in exposed patients. **(B)** SWM cells in exposed or non-exposed HC and SLE patients. **(C)** DN1 and DN2 cells in exposed or non-exposed patients. **(D)** DN2/DN1 ratio in exposed or non-exposed patients. **(E)** SWM cells and exposure to degreasers and ketones in patients. **(F)** USM cells in exposed or non-exposed HC and SLE patients. **(G)** Percentage of exposed inactive and active patients and Mex-SLEDAI index in exposed or not patients. **(H)** Dimensionality reduction analysis in exposed or non-exposed patients. **(I)** Clusters with a differential expression when comparing between exposed or non-exposed patients and its markers. *P ≤ 0.05; **P ≤ 0.01; ***P ≤ 0.001.

We observed significant differences in the distribution of SWM cells between SLE and OS-SLE (Figure 5A). Specifically, exposed patients had an increase in SWM cells (means of 21.4% for OS-SLE vs. 14.1% for SLE, *p* = 0.038) (Figure 5B).

DN1 cells are thought to be precursors of SWM cells through the germinal center differentiation pathway (37), and a trend was observed in the increase of DN1 cells in exposed patients (46.3 vs. 28.1 *p* = 0.054) (Figure 5C).

Exposure could also potentially affect the cells of the extrafollicular pathway but the frequencies of DN2 cells did not differ (Figure 5C), suggesting that solvents could have a possible effect on the differentiation germinal center but not on the extrafollicular pathway. This was further supported by the Ratio of DN2/DN1 (0.456 vs. −9.441 *p* = 0.0456) (Figure 5D).

Further analysis indicated that the frequency of SWM cells was higher in patients exposed to both degreasers (26.6% vs. 14.4% for SLE, *p* = 0.004) and ketones (26.8% for vs. 15.4% for SLE, *p* = 0.037) (Figure 5E) and this association was not influenced by the duration of the disease.

Evidence in murine models has shown an association between activity and solvents; therefore, we asked whether patients exposed to solvents would have more disease activity. A higher proportion of active SLE patients were exposed to OS (50% compared to 26% in the inactive group; Figure 5G). However, group analysis failed to demonstrate significant differences in the distribution of MEX-SLEDAI scores between patients exposed or not to OS (Figure 5G).

Next, we questioned whether exposed healthy controls would have alterations in B cell distribution. While these findings should be considered preliminary based on the small sample size, the two subjects available for analysis demonstrated a striking decrease in USM cells to levels comparable to those seen in SLE patients (Figure 5F).

Finally, we performed dimensionality reduction analysis to identify possible new B cell subsets associated with solvents exposure. Four subsets have markers compatible with SWM cells and were overexpressed in exposed patients (Figures 5H,I), aligning with our results.

## Discussion

**FIGURE 6:**
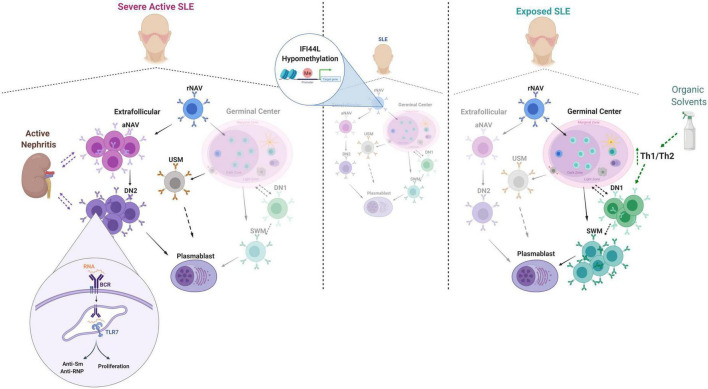
Summary of main findings and discussion elements. The dotted lines denote possible relationships or trends that require further validation studies. This figure was designed with Biorender.com.

The B cell subpopulation profile of Colombian SLE patients differs from that in HC; in line with the literature for other populations of lupus patients (37, 38). We also found distinct distributions of cells of the two differentiation pathways. In the extrafollicular pathway, we observed an expansion of aNAV and DN2 cells associated with severe activity and other clinical features. In the germinal center differentiation pathway, we observed increased frequencies of SWM and DN1 cells in patients exposed to solvents; to our knowledge, this is the first description of this phenomenon. These findings and the main elements of the discussion are summarized in Figure 6.

Our finding of an increase in extrafollicular cells associated with severe disease activity, nephritis, positive Anti-Sm and Anti RNP titers, and decreased C3 could be explained by a possible pathogenetic role of these cells in SLE. Previous evidence has shown hyperactivity to TLR7 (12), and in the presence of IFN, they would be 100 times more sensitive (39). Additionally, DN2 cells can produce autoantibodies (12), which could cause organ damage. Since patients with SLE have defects in removing apoptotic bodies, the availability of RNA-type antigens would increase and stimulate TLR7 again, which could lead to a vicious cycle that could trigger a flare in patients. Actually, in a longitudinal analysis, although it had a limited number of patients, the increases in aNAV cells occurred before and after a flare (40). Therefore, aNAV cells could have value as a predictive biomarker of clinical relapse, but this phenomenon may vary according to ethnicity.

Colombia’s population is highly diverse and it has a mixture of ancestries (41). Ossa et al. described that the percentage of each varies in some areas of the country. For example, in the region near the Amazon, the population has more than 65% of Native-American ancestry genes, while the population near the Pacific coast has around 63% African contribution. SLE Patients recruited in this study were from Medellin city, located in the country’s central region, near the Andes Mountains range. The first Europeans in the country were established here; therefore, the population of this part of the country had 67% of genes of European ancestry, 25% of Native-American ancestry genes, and 7% of African genes (13). Thus, we had hypothesized that the DN2 cell distribution of Mestizo patients in this study would resemble what has been described for European-American patients. Strikingly, DN2 cell distribution in Mestizo SLE patients resembles closer to the reported data of African- American patients, both on frequencies (87% of patients had high DN2 cell frequencies) and in associations with disease activity, nephritis, and anti-Sm and Anti RNP autoantibodies. Consistent with studies in North America, African Colombian patients had the highest frequencies of DN2 cells. Also, Colombian SLE patients showed higher DN2 cells overall when compared to European American patients.

In a study conducted in a cohort of Asian patients with SLE, an expansion of aNAV and DN2 B cells was associated with disease activity (42). The similarity of their clinical associations in a population of patients with Asian ancestry, which also represents the third population in prevalence affected by SLE in the world, suggests a role of B cell extrafollicular differentiation pathway and a possible role in the pathophysiology of SLE, previously described in African-American patients, evidenced in Asian patients and confirmed in this study in Latin American patients. Recent work on the immune response of patients with severe COVID-19 also supports this hypothesis of a possible pathogenic role of extrafollicular cells (43).

One of the most relevant clinical associations of extrafollicular cells aNAV and DN2 was nephritis. To date, the presence of aNAV or DN2 cells characterized by the markers described in the present study has not been described in samples of kidney tissue from patients with lupus nephritis. However, some studies that use different discrimination strategies for B cell subsets that likely correspond to DN2 cells have been described in kidney tissues (44, 45). They described a spectrum of B cells *in situ* that included cells with markers reported on ABC cells (Age-Associated B cells). Regarding ABC cells, these were initially described in animal models as expanded extrafollicular B cells in mice with autoimmunity and characterized by CD11c and T-bet. Recently, it has been proposed that their human equivalents are aNAV and DN2 cells (46). This suggests that the intrarenal ABC cells described by Arazi et al. and Wang et al. may be the same as aNAV and DN2 cells, which would help explain the association found with nephritis. Indeed, a study described the association of atypical memory cells with nephritis also detected intrarenal CD20+, T-bet+B cells from Asian patients with SLE (47).

We also found a novel association between USM cells and thrombosis. B cell dysregulation has been reported in patients with antiphospholipid syndrome (APS), with decreased USM, especially in patients with thrombosis (48). Although the pathophysiology of B cell dysregulation on APS is not known, associated APS is a clinical manifestation of SLE, and this association could reflect the APS effect on B cells. Also, decreased USM has been attributed to deficient spleen function (49). Indeed, Hyposplenism has been described in SLE (50, 51), and there are also reports of SLE patients with secondary APS with autosplenectomy caused by spleen infarcts (52). This could explain the decrease in USM seen in patients with a history of thrombosis (Figure 2B).

Most interestingly, both the reversible decrease in USM and the recovery of splenic deficiency have been observed in Inflammatory Bowel Disease (IBD) patients treated with anti-TNF (53). Therefore, hyposplenism seems to be a common mechanism on SLE, secondary APS, and IBD that could relate to low USM cells. However, spleen deficiency could be explained by different physiopathological mechanisms in these diseases. For APS, spleen infarcts could be the cause; for IBD, TNF seems to play a role, and SLE inflammation could be the key. Although TNF involvement in SLE needs more research, reports on drug-induced SLE after anti-TNF treatment (54) could suggest a different mechanism for spleen deficiency. On other possibilities, USM cells are a marginal-zone equivalent (55) that includes Regulatory B cells (Bregs) (56), and therefore their decrease might correlate with impaired B regulatory function.

The present study found hypomethylation of the two IFI44L gene promoter sites in the patients’ rNAV cell subpopulation. This agrees with the literature regarding the methylation status of the IFI44L gene in SLE, of which its hypomethylation has been reported in total B cells, CD4 T lymphocytes, monocytes, neutrophils, and PBMC (57). Finding this change in rNAV cells suggests that it could also be found in other cells in later stages of differentiation, given the epigenetic imprinting described in SLE patients (35). On the other hand, these methylation patterns are specific according to ethnicity (58). In this case, this is the first study to analyze this gene in Latin American patients with SLE, and the hypomethylation we found is again more similar to that reported for African American patients (59).

B cell dysregulation could be explained by several factors, including epigenetic changes and environmental exposure. To our knowledge, this is the first analysis of the association and possible influence of OS exposure on B cell regulation in SLE. Although epidemiological studies have not been conclusive on OS as a risk factor for SLE (60), there is enough evidence to postulate such a role based on autoimmune responses in animal models and the effects on SLE progression caused by exposure to TCE (61). Of importance for our findings, degreasers were the main OS to which both HC and SLE patients in this study were exposed. This finding, therefore, indicates the need for further research on the role of OS in human autoimmunity and specifically in SLE.

Systemic lupus erythematosus activity was not associated with OS exposure, but we noted a trend where patients with high disease activity had more OS exposure. In line with this, Li et al. found a higher risk of hospitalization among SLE patients with occupations related to OS exposure, like artistic workers and shoe and leather workers (62).

The most consistent difference observed between patients exposed or not exposed to OS was a higher frequency of CD27+ SWM B cells in exposed patients. While the loss of USW that we and others have reported is common to all lupus patients, and effector (12), activated (63), and antibody-secreting cells (40) are elevated in specific patient subgroups, patients with a predominance of SWM have been little studied. It is presently unclear how OS exposure may favor the development of SWM. CD27+ SWM is thought to be the product of T/B interactions during germinal center reactions (64). The influence of OS on SWM differentiation may be mediated through CD4 T cells. Occupational exposure to TCE results in reduced B and T lymphocytes, decreased IL10 (36), IL4, and TNF-a, and increased IL2 and INF- γ (65). It is essential to note that this is in healthy individuals and may be different in the context of lupus; supporting this, we observed a reduction in neither lymphocytes nor CD19 + B cells in OS-exposed lupus patients (Supplementary Figure 4). Mouse lupus models exposed to Trichloroethylene (TCE) have increased T cell activation, particularly on CD4 + T cells expressing activation markers and increased secretion of INF-γ (20); also, a recent study on BALB/c mice exposed to TCE proposes a tendency of T cells to differentiate toward a Th1 profile, therefore altering the Th1/Th2 ratio (66). However, T cell phenotyping in human exposure studies has been limited and not done in autoimmune patients. Increased interferon-gamma signaling causes excessive T follicular helper development and germinal center formation in some mouse models (67), and a similar mechanism may operate in OS-exposed lupus patients. This is also supported by Kaneko et al.; they described an immunoblastic cell-like structure in the spleen of MRL-*lpr/lpr* mice exposed to TCE, suggesting an inclination to form germinal centers (68).

A complementary process may be the development of tertiary lymphoid organs in tissues at the site of exposure. Indeed, studies on MRL+ mice exposed to TCE have shown lymphocytic infiltration on the skin and resultant alopecia (19), as well as a lymphocytic infiltration on kidneys, lungs, and livers (69) of chronically exposed mice. Interestingly, while DN2 B cells, were expanded in some patients, these cells were not associated with OS exposure even though a substantial portion of these patients have active disease. Potentially, OS may result in more germinal center-derived pathology in contrast to the extra-follicular response found in other patients (46). For example, ASC from OS-exposed patients may have more somatic hypermutations and increased affinity maturation.

Longitudinal studies could be recommended further validate this association. Further research on this association between environmental factors and B cell subsets could contribute to the understanding of flares prevention, which could improve outcomes in SLE patients. The observation that OS exposure may play a role in B cell dysregulation and possibly, the development of clinical autoimmunity bears multiple implications and open the door to epidemiological, clinical, and immunological investigations, including a dissection of the signaling pathways and molecular programs triggered by these agents. Indeed, as an environmental factor, OS could induce epigenetic changes in genes that orchestrate the B cell differentiation process, as currently demonstrated for SLE B cells (14).

The scope of the results of this study must be analyzed within the framework of its limitations. The use of frozen cells did not allow us to characterize ASC cells. It should also be considered that it was not possible to measure the presence of chemical substances in the patients’ blood for a thorough characterization of environmental exposure. Also, the main limitation of this study lies in the sample size, which restricts the scope to an exploratory approach in the analysis of environmental exposure. Finally, other environmental factors that could play a role in SLE, such as nutritional deficiencies of vitamins B12, and B6 or exposure to mercury, were not measured.

## Data availability statement

The original contributions presented in this study are included in the article/Supplementary material, further inquiries can be directed to the corresponding author.

## Ethics statement

The Ethics Committee on Research on Human Beings of CES University with the record number 118 and the Emory Institutional Review Board (58515 and 58507) approved the study. The patients/participants provided their written informed consent to participate in this study.

## Author contributions

CH, SJ, GV, and IS: conceptualization and writing—original draft. CH, KC, JD-C, SJ, and GV: investigation. CH, DR-G, RU, EV-T, MR, and SJ: analysis. CH: visualization. SJ, GV, and IS: supervision. GV and IS: funding acquisition and resources. All authors methodology, writing—review, and approved the submitted version.

## Abbreviations

NAV: Naive CD27^–^IgD^+^
DN: double negative CD27^–^IgD^–^
rNAV: resting Naive CD21^+^ CD11c^–^
aNAV: active Naive CD21^–^ CD11c^+^
DN1: double negative type 1 CD21^+^ CD11c^–^
DN2: double negative type 2 CD21^–^ CD11c^+^
SWM: switched memory CD27^+^IgD^–^
USM: unswitched memory CD27^+^IgD^+^
TCE: trichloroethylene
OS: organic solvents.

## References

[1] Lockshin MD Barbhaiya M Izmirly P Buyon JP Crow MK. SLE: reconciling heterogeneity. *Lupus Sci Med.* (2019) 6:e000280. 10.1136/lupus-2018-000280 31080630 PMC6485210

[2] Kaul A Gordon C Crow MK Touma Z Urowitz MB van Vollenhoven R Systemic lupus erythematosus. *Nat Rev Dis Primers.* (2016) 2:16039. 10.1038/nrdp.2016.39 27306639

[3] Györi N Giannakou I Chatzidionysiou K Magder L van Vollenhoven RF Petri M. Disease activity patterns over time in patients with SLE: analysis of the Hopkins lupus cohort. *Lupus Sci Med.* (2017) 4:e000192. 10.1136/lupus-2016-000192 PMC5307372 28243457

[4] Fanouriakis A Bertsias G. Changing paradigms in the treatment of systemic lupus erythematosus. *Lupus Sci Med.* (2019) 6:e000310. 10.1136/lupus-2018-000310 31168398 PMC6519431

[5] Pons-Estel GJ Alarcón GS Scofield L Reinlib L Cooper GS. Understanding the epidemiology and progression of systemic lupus erythematosus. *Semin Arthrit Rheumat.* (2010) 39:257–68. 10.1016/j.semarthrit.2008.10.007 19136143 PMC2813992

[6] Pons-Estel BA Catoggio LJ Cardiel MH Soriano ER Gentiletti S Villa AR The GLADEL multinational Latin American prospective inception cohort of 1,214 patients with systemic lupus erythematosus: ethnic and disease heterogeneity among “Hispanics.”. *Medicine.* (2004) 83:1–17. 10.1097/01.md.0000104742.42401.e2 14747764

[7] Pons-Estel GJ Catoggio LJ Cardiel MH Bonfa E Caeiro F Sato E Lupus in Latin-American patients: lessons from the GLADEL cohort. *Lupus.* (2015) 24:536–45. 10.1177/0961203314567753 25697768

[8] Lewis MJ Jawad AS. The effect of ethnicity and genetic ancestry on the epidemiology, clinical features and outcome of systemic lupus erythematosus. *Rheumatology.* (2016) 56:67–77. 10.1093/rheumatology/kew399 27940583

[9] Mejía-Vilet JM Córdova-Sánchez BM Arreola-Guerra JM Morales-Buenrostro LE Uribe-Uribe NO Correa-Rotter R. Renal flare prediction and prognosis in lupus nephritis Hispanic patients. *Lupus.* (2016) 25:315–24. 10.1177/0961203315606985 26405028

[10] Sanz I Lee FE-H. B cells as therapeutic targets in SLE. *Nat Rev Rheumatol.* (2010) 6:326–37. 10.1038/nrrheum.2010.68 20520647 PMC3934759

[11] Wei C Jenks S Sanz I. Polychromatic flow cytometry in evaluating rheumatic disease patients. *Arthrit Res Ther.* (2015) 17:46. 10.1186/s13075-015-0561-1 25880288 PMC4350283

[12] Jenks SA Cashman KS Zumaquero E Marigorta UM Patel AV Wang X Distinct effector B cells induced by unregulated toll-like receptor 7 contribute to pathogenic responses in systemic lupus erythematosus. *Immunity.* (2018) 49:725.e–39.e. 10.1016/j.immuni.2018.08.015 30314758 PMC6217820

[13] Ossa H Aquino J Pereira R Ibarra A Ossa RH Pérez LA Outlining the ancestry landscape of colombian admixed populations. *PLoS One.* (2016) 11:e0164414. 10.1371/journal.pone.0164414 27736937 PMC5063461

[14] Long H Yin H Wang L Gershwin ME Lu Q. The critical role of epigenetics in systemic lupus erythematosus and autoimmunity. *J Autoimmun.* (2016) 74:118–38. 10.1016/j.jaut.2016.06.020 27396525

[15] Wiley KL Treadwell E Manigaba K Word B Lyn-Cook BD. Ethnic differences in DNA methyltransferases expression in patients with systemic lupus erythematosus. *J Clin Immunol.* (2013) 33:342–8. 10.1007/s10875-012-9803-z 23054340 PMC3573322

[16] Joseph S George NI Green-Knox B Treadwell EL Word B Yim S Epigenome-wide association study of peripheral blood mononuclear cells in systemic lupus erythematosus: identifying DNA methylation signatures associated with interferon-related genes based on ethnicity and SLEDAI. *J Autoimmun.* (2018) 96:147–157. 10.1016/j.jaut.2018.09.007 30301579

[17] Zhao M Zhou Y Zhu B Wan M Jiang T Tan Q IFI44L promoter methylation as a blood biomarker for systemic lupus erythematosus. *Ann Rheumat Dis.* (2016) 75:1998–2006. 10.1136/annrheumdis-2015-208410 26787370 PMC4955646

[18] Draborg AH Duus K Houen G. Epstein-barr virus and systemic lupus erythematosus. *Clin Dev Immunol.* (2012) 2012:1–10. 10.1155/2012/370516 22811739 PMC3395176

[19] Blossom SJ Doss JC Gilbert KM. Chronic exposure to a trichloroethylene metabolite in autoimmune-prone MRL+/+ mice promotes immune modulation and alopecia. *Toxicol Sci.* (2006) 95:401–11. 10.1093/toxsci/kfl149 17077186

[20] Wang G Wang J Ma H Khan MF. Increased nitration and carbonylation of proteins in MRL +/+ mice exposed to trichloroethene: potential role of protein oxidation in autoimmunity. *Toxicol Appl Pharmacol.* (2009) 237:188–95. 10.1016/j.taap.2009.03.010 19332086 PMC2734328

[21] Guzmán J Cardiel MH Arce-Salinas A Sánchez-Guerrero J Alarcón-Segovia D. Measurement of disease activity in systemic lupus erythematosus. Prospective validation of 3 clinical indices. *J Rheumatol.* (1992) 19:1551–8. 1464867

[22] Uribe AG Vilá LM McGwin G Sanchez ML Reveille JD Alarcón GS. The systemic lupus activity measure-revised, the Mexican systemic lupus erythematosus disease activity index (SLEDAI), and a modified SLEDAI-2K are adequate instruments to measure disease activity in systemic lupus erythematosus. *J Rheumatol.* (2004) 31:1934–40. 15468356

[23] Salazar-Camarena DC Ortiz-Lazareno PC Cruz A Oregon-Romero E Machado-Contreras JR Muñoz-Valle JF Association of BAFF, APRIL serum levels, BAFF-R, TACI and BCMA expression on peripheral B-cell subsets with clinical manifestations in systemic lupus erythematosus. *Lupus.* (2016) 25:582–92. 10.1177/0961203315608254 26424128

[24] Kaminski DA Wei C Rosenberg AF Lee FE-H Sanz I. Multiparameter flow cytometry and bioanalytics for b cell profiling in systemic lupus erythematosus. In: Perl A editor. *Autoimmunity.* (Vol. 900), Totowa, NJ: Humana Press (2012). p. 109–34. 10.1007/978-1-60761-720-4_6 PMC3927893 22933067

[25] Jenks S Marcus J Cashman K Sanz I. B cell binding autoreactive VH4.34 antibodies are speciõc to lupus, consist of diverse isotypes, and are associated with high disease activity and lupus nephritis [abstract]. *Arthritis Rheumatol.* (2017) 69:1–2.

[26] Hurtado C Rojas-Gualdrón D Hernández D Díaz JC Urrego R Vásquez-Trespalacios EM Construction of a questionnaire to characterize environmental exposures in patients with systemic lupus erythematosus. *Rev Colomb Reumatol (English Edition).* (2021) 28:255–66. 10.1016/j.rcreue.2020.07.006

[27] Genser B Cooper PJ Yazdanbaksh M Barreto ML Rodrigues LC. A guide to modern statistical analysis of immunological data. *BMC Immunol.* (2007) 8:27. 10.1186/1471-2172-8-27 17963513 PMC2234437

[28] Isenberg D Spellerberg M Williams W Griffiths M Stevenson F. Identification of the 9G4 idiotope in systemic lupus erythematosus. *Rheumatology.* (1993) 32:876–82. 10.1093/rheumatology/32.10.876 7691367

[29] Anolik JH Barnard J Cappione A Pugh-Bernard AE Felgar RE Looney RJ Rituximab improves peripheral B cell abnormalities in human systemic lupus erythematosus. *Arthritis Rheumat.* (2004) 50:3580–90. 10.1002/art.20592 15529346

[30] Richardson C Chida AS Adlowitz D Silver L Fox E Jenks SA Molecular basis of 9G4 B cell autoreactivity in human systemic lupus erythematosus. *J Immunol.* (2013) 191:4926–39. 10.4049/jimmunol.1202263 24108696 PMC3816606

[31] Cappione A Anolik JH Pugh-Bernard A Barnard J Dutcher P Silverman G Germinal center exclusion of autoreactive B cells is defective in human systemic lupus erythematosus. *J Clin Invest.* (2005) 115:3205–16. 10.1172/JCI24179 16211091 PMC1242189

[32] Mair F Hartmann FJ Mrdjen D Tosevski V Krieg C Becher B. The end of gating? An introduction to automated analysis of high dimensional cytometry data: highlights. *Eur J Immunol.* (2016) 46:34–43. 10.1002/eji.201545774 26548301

[33] Roberts MEP Kaminski D Jenks SA Maguire C Ching K Burbelo PD Primary sjögren’s syndrome is characterized by distinct phenotypic and transcriptional profiles of IgD+ unswitched memory b cells: memory b cell phenotype and gene expression in sjögren’s syndrome. *Arthritis Rheumatol.* (2014) 66:2558–69. 10.1002/art.38734 24909310 PMC4160119

[34] Chen SH Lv QL Hu L Peng MJ Wang GH Sun B. DNA methylation alterations in the pathogenesis of lupus: DNA methylation alterations in lupus. *Clin Exp Immunol.* (2017) 187:185–92. 10.1111/cei.12877 27690369 PMC5217979

[35] Scharer CD Blalock EL Mi T Barwick BG Jenks SA Deguchi T Epigenetic programming underpins B cell dysfunction in human SLE. *Nat Immunol.* (2019) 20:1071–82. 10.1038/s41590-019-0419-9 31263277 PMC6642679

[36] Bassig BA Zhang L Tang X Vermeulen R Shen M Smith MT Occupational exposure to trichloroethylene and serum concentrations of IL-6, IL-10, and TNF-alpha: trichloroethylene exposure and serum cytokine levels. *Environ Mol Mutagenesis.* (2013) 54:450–4. 10.1002/em.21789 23798002 PMC4360987

[37] Sanz I Wei C Jenks SA Cashman KS Tipton C Woodruff MC Challenges and opportunities for consistent classification of human B cell and plasma cell populations. *Front Immunol.* (2019) 10:2458. 10.3389/fimmu.2019.02458 31681331 PMC6813733

[38] Jenks SA Wei C Bugrovsky R Hill A Wang X Rossi FM B cell subset composition segments clinically and serologically distinct groups in chronic cutaneous lupus erythematosus. *Ann Rheum Dis.* (2021) 80:1190–200. 10.1136/annrheumdis-2021-220349 34083207 PMC8906255

[39] Zumaquero E Stone SL Scharer CD Jenks SA Nellore A Mousseau B IFNγ induces epigenetic programming of human T-bethi B cells and promotes TLR7/8 and IL-21 induced differentiation. *ELife.* (2019) 8:e41641. 10.7554/eLife.41641 31090539 PMC6544433

[40] Tipton CM Fucile CF Darce J Chida A Ichikawa T Gregoretti I Diversity, cellular origin and autoreactivity of antibody-secreting cell population expansions in acute systemic lupus erythematosus. *Nat Immunol.* (2015) 16:755–65. 10.1038/ni.3175 26006014 PMC4512288

[41] Mooney JA Huber CD Service S Sul JH Marsden CD Zhang Z Understanding the hidden complexity of Latin American population isolates. *Am J Hum Genet.* (2018) 103:707–26. 10.1016/j.ajhg.2018.09.013 30401458 PMC6218714

[42] Wangriatisak K Thanadetsuntorn C Krittayapoositpot T Leepiyasakulchai C Suangtamai T Ngamjanyaporn P The expansion of activated naive DNA autoreactive B cells and its association with disease activity in systemic lupus erythematosus patients. *Arthritis Res Ther.* (2021) 23:179. 10.1186/s13075-021-02557-0 34229724 PMC8259008

[43] Woodruff MC Ramonell RP Nguyen DC Cashman KS Saini AS Haddad NS Extrafollicular B cell responses correlate with neutralizing antibodies and morbidity in COVID-19. *Nat Immunol.* (2020) 21:1506–16. 10.1038/s41590-020-00814-z 33028979 PMC7739702

[44] Arazi A Rao DA Berthier CC Davidson A Liu Y Hoover PJ The immune cell landscape in kidneys of patients with lupus nephritis. *Nat Immunol.* (2019) 20:902–14. 10.1038/s41590-019-0398-x 31209404 PMC6726437

[45] Wang S Wang J Kumar V Karnell JL Naiman B Gross PS IL-21 drives expansion and plasma cell differentiation of autoreactive CD11chiT-bet+ B cells in SLE. *Nat Commun.* (2018) 9:1758. 10.1038/s41467-018-03750-7 29717110 PMC5931508

[46] Jenks SA Cashman KS Woodruff MC Lee FE Sanz I. Extrafollicular responses in humans and SLE. *Immunol Rev.* (2019) 288:136–48. 10.1111/imr.12741 30874345 PMC6422038

[47] Wu C Fu Q Guo Q Chen S Goswami S Sun S Lupus-associated atypical memory B cells are mTORC1-hyperactivated and functionally dysregulated. *Ann Rheumat Dis.* (2019) 78:1090–100. 10.1136/annrheumdis-2019-215039 31142473 PMC6691860

[48] Álvarez-Rodríguez L Riancho-Zarrabeitia L Calvo-Alén J López-Hoyos M Martínez-Taboada V. Peripheral B-cell subset distribution in primary antiphospholipid syndrome. *Int J Mol Sci.* (2018) 19:589. 10.3390/ijms19020589 29462939 PMC5855811

[49] Lammers AJJ de Porto APNA Bennink RJ van Leeuwen EMM Biemond BJ Goslings JC Hyposplenism: comparison of different methods for determining splenic function. *Am J Hematol.* (2012) 87:484–9. 10.1002/ajh.23154 22488175

[50] Childs JC Adelizzi RA Dabrow MB Freed N. Splenic hypofunction in systemic lupus erythematosus. *J Am Osteopath Assoc.* (1994) 94:414–5. 8056631

[51] Sánchez-Guerrero SA Sánchez-Guerrero J. Persistent thrombocytosis in systemic lupus erythematosus. Activity, Reactivity, or what? *J Rheumatol.* (2007) 34:1441–2. 17611959

[52] Santilli D Govoni M Prandini N Rizzo N Trotta F. Autosplenectomy and antiphospholipid antibodies in systemic lupus erythematosus: a pathogenetic relationship? *Semin Arthritis Rheum.* (2003) 33:125–33. 10.1016/s0049-0172(03)00004-0 14625820

[53] Di Sabatino A Rosado MM Cazzola P Biancheri P Tinozzi FP Laera MR Splenic function and IgM-memory B cells in Crohn’s disease patients treated with infliximab. *Inflammatory Bowel Dis.* (2008) 14:591–6. 10.1002/ibd.20374 18240280

[54] Shovman O Tamar S Amital H Watad A Shoenfeld Y. Diverse patterns of anti-TNF-α-induced lupus: case series and review of the literature. *Clin Rheumatol.* (2018) 37:563–8. 10.1007/s10067-017-3884-2 29063464

[55] Weill J-C Weller S Reynaud C-A. Human marginal zone B cells. *Annu Rev Immunol.* (2009) 27:267–85. 10.1146/annurev.immunol.021908.132607 19302041

[56] Wortel C Heidt S. Regulatory B cells: phenotype, function and role in transplantation. *Transplant Immunol.* (2017) 41:1–9. 10.1016/j.trim.2017.02.004 28257995

[57] Hurtado C Acevedo Sáenz LY Vásquez Trespalacios EM Urrego R Jenks S Sanz I DNA methylation changes on immune cells in systemic lupus erythematosus. *Autoimmunity.* (2020) 53:114–21. 10.1080/08916934.2020.1722108 32019373 PMC8063264

[58] Ballestar E Sawalha AH Lu Q. Clinical value of DNA methylation markers in autoimmune rheumatic diseases. *Nat Rev Rheumatol.* (2020) 16:514–24. 10.1038/s41584-020-0470-9 32759997 PMC7955859

[59] Breitbach ME Ramaker RC Roberts K Kimberly RP Absher D. Population-specific patterns of epigenetic defects in the B cell lineage in patients with systemic lupus erythematosus. *Arthritis Rheumatol.* (2020) 72:282–91. 10.1002/art.41083 31430064

[60] Parks C De Roos A. Pesticides, chemical and industrial exposures in relation to systemic lupus erythematosus. *Lupus.* (2014) 23:527–36. 10.1177/0961203313511680 24763537 PMC4020505

[61] Cooper GS Makris SL Nietert PJ Jinot J. Evidence of autoimmune-related effects of trichloroethylene exposure from studies in mice and humans. *Environ Health Perspect.* (2009) 117:696–702. 10.1289/ehp.11782 19479009 PMC2685829

[62] Li X Sundquist J Sundquist K Zöller B. Occupational risk factors for systemic lupus erythematosus: a nationwide study based on hospitalizations in Sweden. *J Rheumatol.* (2012) 39:743–51. 10.3899/jrheum.110789 22382347

[63] Jacobi AM Reiter K Mackay M Aranow C Hiepe F Radbruch A Activated memory B cell subsets correlate with disease activity in systemic lupus erythematosus: delineation by expression of CD27, IgD, and CD95. *Arthritis Rheumat.* (2008) 58:1762–73. 10.1002/art.23498 18512812

[64] Berkowska MA Driessen GJA Bikos V Grosserichter-Wagener C Stamatopoulos K Cerutti A Human memory B cells originate from three distinct germinal center-dependent and -independent maturation pathways. *Blood.* (2011) 118:2150–8. 10.1182/blood-2011-04-345579 21690558 PMC3342861

[65] Iavicoli I Marinaccio A Carelli G. Effects of occupational trichloroethylene exposure on cytokine levels in workers. *J Occup Environ Med.* (2005) 47:453–7. 10.1097/01.jom.0000161728.23285.66 15891523

[66] Li S Yu Y Yang P Wang H Zhang C Liu M Trichloroethylene alters Th1/Th2/Th17/Treg paradigm in mice: a novel mechanism for chemically induced autoimmunity. *Int J Toxicol.* (2018) 37:155–63. 10.1177/1091581818757036 29554824 PMC11951856

[67] Lee SK Silva DG Martin JL Pratama A Hu X Chang P-P Interferon-γ excess leads to pathogenic accumulation of follicular helper T cells and germinal centers. *Immunity.* (2012) 37:880–92. 10.1016/j.immuni.2012.10.010 23159227

[68] Kaneko T Saegusa M Tasaka K Sato A. Immunotoxicity of trichloroethylene: a study with MRL-lpr/lpr mice. *J Appl Toxicol.* (2000) 20:471–5. 10.1002/1099-1263(200011/12)20:6<471::aid-jat716>3.0.co;2-e 11180269

[69] Cai P König R Boor PJ Kondraganti S Kaphalia BS Khan MF Chronic exposure to trichloroethene causes early onset of SLE-like disease in female MRL +/+ mice. *Toxicol Appl Pharmacol.* (2008) 228:68–75. 10.1016/j.taap.2007.11.031 18234256 PMC2442272

