# Supplementary material for: Altered B cell phenotype and CD27+ memory B cells are associated with clinical features and environmental exposure in Colombian systemic lupus erythematosus patients
Source: Front Med (Lausanne). 2022 Sep 6;9:950452. doi: 10.3389/fmed.2022.950452 (PMC9485945; doi:10.3389/fmed.2022.950452)
Supplement: Supplementary file 1 [file Data_Sheet_1.pdf]

Supplementary Material

Supplementary Table 1. Demographic and Clinical characteristics of participants

		Active n = 10 n (%)	Inactive n = 30 n (%)	HC n=17 n (%)	p-value
**Gender**	Female	10 (100)	27 (90)	15 (88)	0.694
**Age**	mean (SD)	33 (10)	40 (15)	36 (10)	0.254
**Ethnicity**	Mestizo - Colombian	8 (80)	27 (90)	17 (100)	0.143
	African - Colombian	2 (20)	3 (10)	0	
**Clinical characteristics**	Disease duration mean (SD)	4.6 (3)	8.83 (11)		0.236
	Mex-SLEDAI score mean (SD)	10.3 (3)	2.5 (2)		<0.001
**History of Organ Damage**	Joint involvement	7 (70)	24 (80)		0.399
	Cutaneous involvement	10 (100)	23 (76.7)		0.109
	Thrombosis event	1 (10)	4 (13)		0.633
	Renal involvement	8 (80)	14 (46.7)		0.069
	Neurological involvement	2 (20)	2 (6.7)		0.256
	Hematological involvement	6 (60)	15 (50)		0.429
	Raynaud	2 (20)	8 (26.7)		0.515
	Vasculitis	2 (20)	1 (3.3)		0.149
**Medications**	Prednisolone	9 (90)	21 (70)		0.204
	Chloroquine	8 (80)	24 (80)		0.659
	Cyclophosphamide	3 (30)	1 (3.3)		0.042
	Azathioprine	2 (20)	9 (30)		0.432
	Mycophenolate	3 (30)	6 (20)		0.399

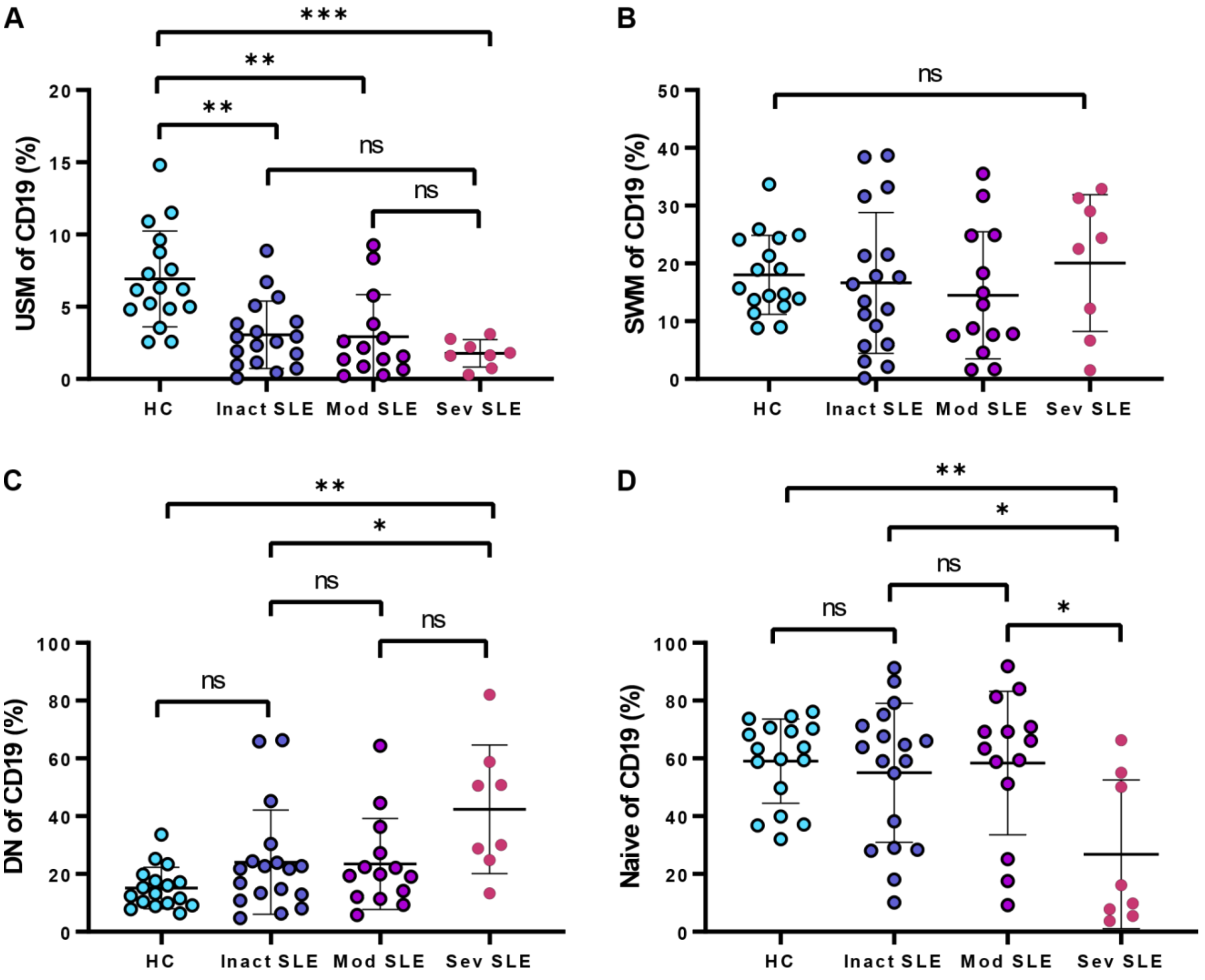

Supplementary figure 1. (A) Unswitched Memory cells (USM), **(B)** Switched Memory (SWM), **(C)** Double Negative (DN), and **(D)** Naive B cells in Healthy Controls (clear blue), Inactive (dark blue), Moderate (purple), and Severe (dark pink) active SLE patients. Multivariate Analysis of Variance (MANOVA) was used for analysis and means, and SD are depicted in the figures.

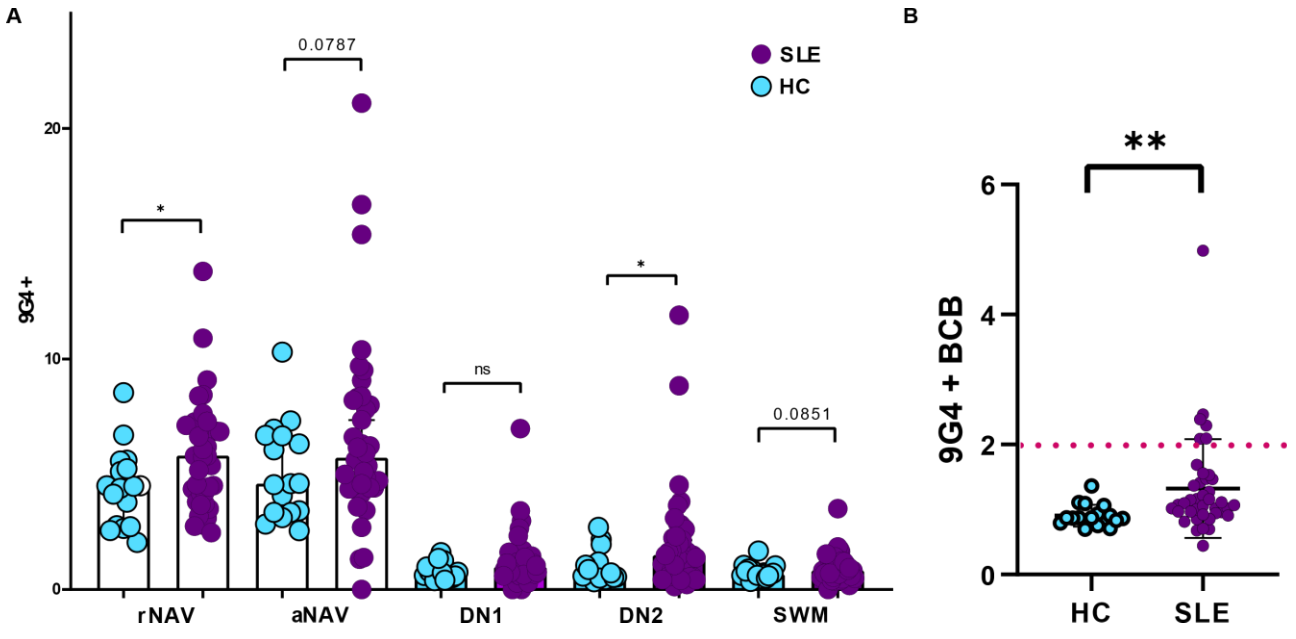

Supplementary figure 2: 9G4 + positive autoreactive B cells. (A). Subpopulations of self-reactive B cells by positivity for 9G4, in patients with SLE and Healthy Controls (**B).** B cell binding (BCB) calculation, with the formula: [Resting naive 9G4$^+$ median fluorescence intensity] / [SWM 9G4$^+$ median fluorescence intensity]. The Mann-Whitney t-test was used for analysis, and the median and 95% CI are plotted in the figures.

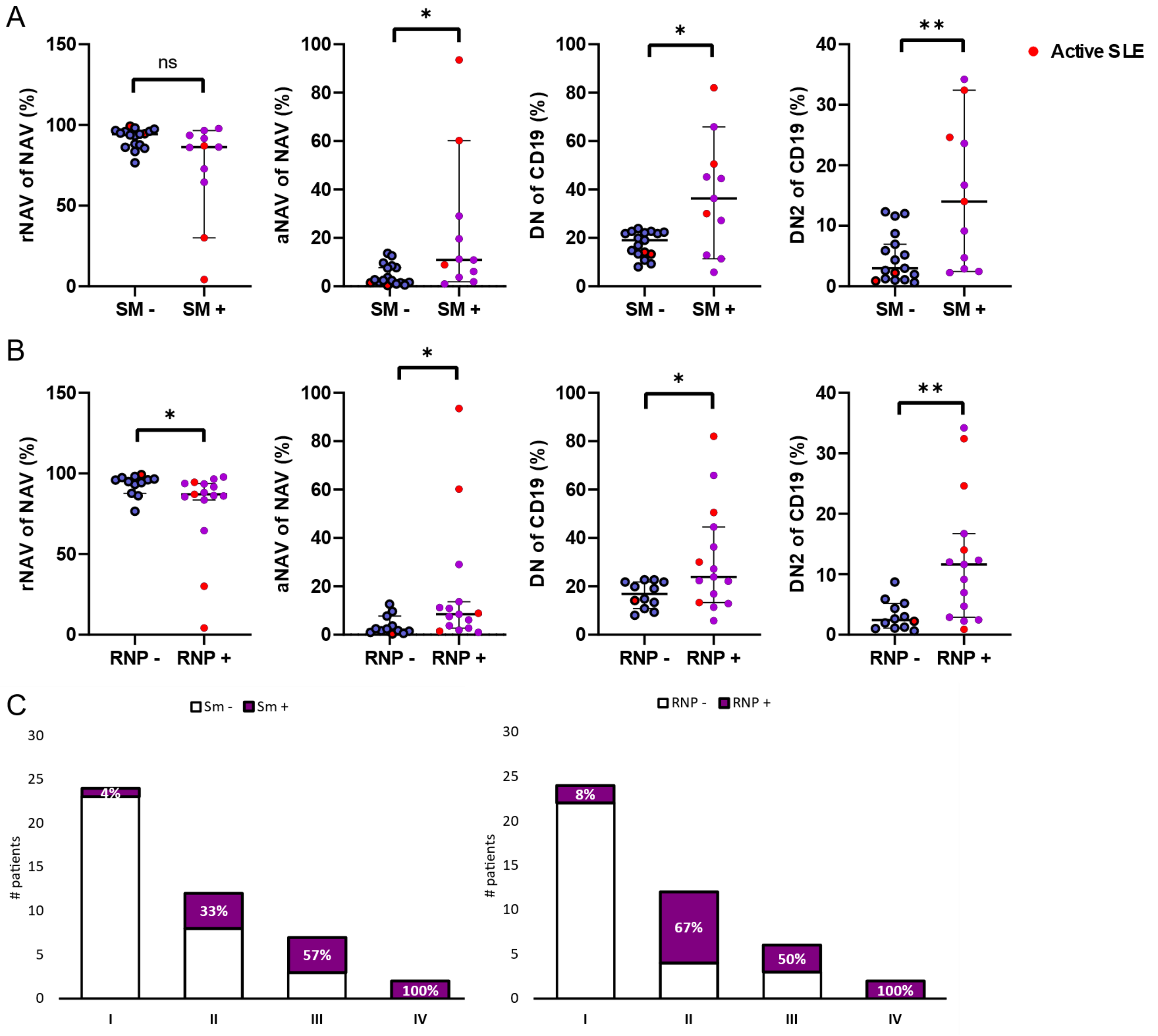

Supplementary figure 3: B cells distribution and autoantibodies (A). B cells subsets associated to Anti Sm autoantibodies (**B).** B cells subsets associated to Anti RNP autoantibodies **(C).** Clinical characteristics associated with clusters in Figure 1E: such as anti-Sm antibodies and anti-RNP; the Chi-square test was used for this analysis.

Supplementary Table 2. Multivariate analysis of associated factors to B cell subsets

Subpoblación	Variable	Log Coef.	P value	95% Conf. Interval	
**Naive of CD19 (%)**	**Severe activity**	-0.772	0.008	-1.331	-0.213
	Hematological involvement	-0.219	0.02	-0.401	-0.037
	Thrombosis	-0.335	0.024	-0.623	-0.047
	Mycophenolate	-0.400	0.004	-0.660	-0.140
**aNAV of NAV (%)**	**Severe activity**	1.151	0.037	0.075	2.227
	Hematological involvement	0.358	0.045	0.008	0.709
	Mycophenolate	0.685	0.009	0.184	1.185
**rNAV of NAV (%)**	Cyclophosphamide	-0.358	0.014	-0.636	-0.079
**DN of CD19 (%)**	Thrombosis	0.339	0.012	0.079	0.600
	Cyclophosphamide	0.354	0.037	0.023	0.684
	Mycophenolate	0.381	0.002	0.146	0.616
**DN2 of DN (%)**	**Ethnicity**	0.294	0.047	0.004	0.584
	Hematological involvement	0.199	0.039	0.010	0.387
**USM of CD19 (%)**	**Thrombosis**	-0.593	0.013	-1.054	-0.132

Adjusted by ethnicity, disease activity, renal involvement, hematological involvement, treatment with cyclophosphamide, and mycophenolate. Only statistically significant results are shown.

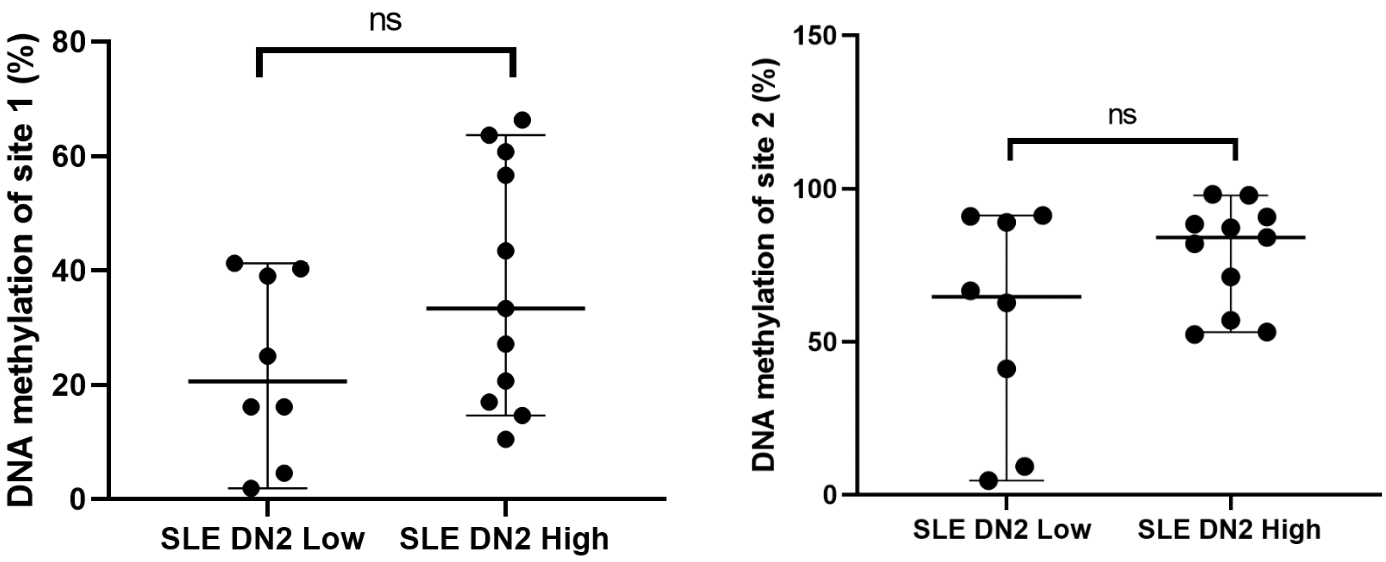

Supplementary figure 4. Percentage of DNA methylation of sites 1 and 2 on the promoter of the gene IFI44L in SLE patients with DN2 high or low frequencies.

Supplementary Table 3. Type of Organic Solvent Exposure

		Patients<br>n=40	HC<br>n=17
		n (%)	n (%)
**Type of Organic Solvent exposure**	Varsol	1 (2.5)	0
	Gasoline	2 (5)	0
	**Ketones**	**4 (10)**	0
	Paintings	1 (2.5)	0
	Paint solvents	1 (2.5)	0
	**Degreasers**	**7 (17.5)**	2 (11.7)
**Use of protection**	**Gloves or mask**	**6 (46.1)**	0

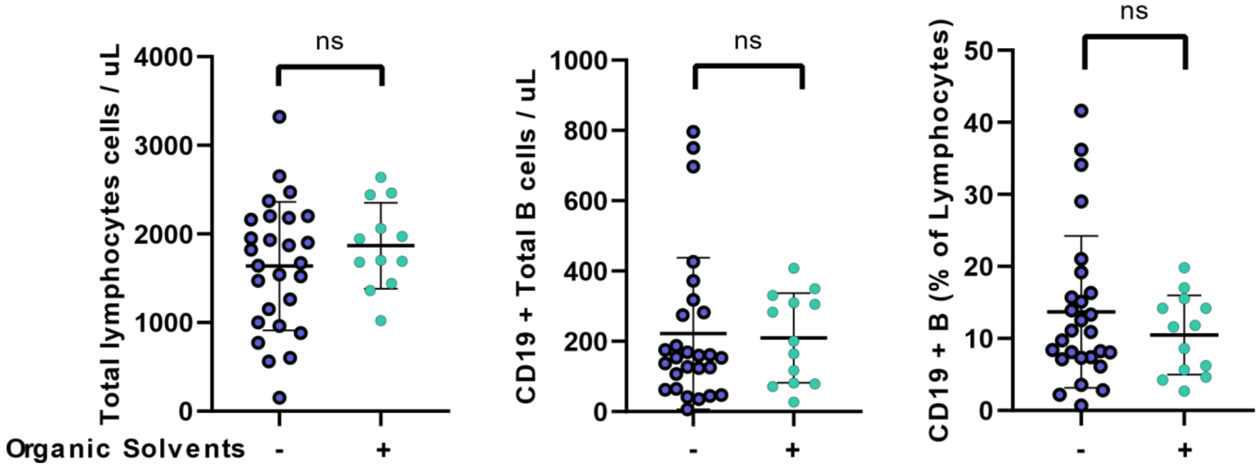

Supplementary figure 5. Total lymphocytes and CD19 + B cells in exposed or non-exposed patients.
